# Feasibility and measurement error in using food supply data to estimate diet costs in Canada

**DOI:** 10.1017/S1368980022000532

**Published:** 2022-06

**Authors:** Gabriella Luongo, Valerie Tarasuk, Yanqing Yi, Catherine L Mah

**Affiliations:** 1 School of Health Administration, Faculty of Health, Dalhousie University, Sir Charles Tupper Medical Building, 5850 College Street, 2nd Floor, PO Box 15000, Halifax, NS B3H 4R2, Canada; 2 Department of Nutritional Sciences, Temerty Faculty of Medicine, University of Toronto, Toronto, ON, Canada; 3 Division of Community Health and Humanities, Faculty of Medicine, Memorial University of Newfoundland, Newfoundland and Labrador, Canada; 4 Dalla Lana School of Public Health, University of Toronto, Toronto, ON, Canada

**Keywords:** Dietary intake, Food prices, Diet cost, Staple foods, Consumer Price Index, Canadian Community Health Survey, Canada

## Abstract

**Objective:**

The cost of food is a key influence on diet. The majority of diet cost studies match intake data from population-based surveys to a single source of food supply prices. Our aim was to examine the methodological significance of using food supply data to price dietary intakes.

**Methods:**

Nationally representative 24-h dietary recall data from the 2015 Canadian Community Health Survey-Nutrition (CCHS-N) was matched to the 2015 Canadian Consumer Price Index (CPI) food price list. Proportions and means of reported intakes covered by the 2015 CPI price list were used to compare reported intakes of food groups and food components of interest and concern overall and by quartile of CPI coverage.

**Setting:**

Canada.

**Participants:**

In total, 20 487 Canadians ages one and older.

**Results:**

The CPI covered on average 76·3 % of total dietary intake (g) without water. Staple food groups that were more commonly consumed had better CPI price coverage than those less commonly consumed. Yet some food groups (vegetables, additions and sweets) that were also commonly consumed by Canadians were not well covered by price data. Individuals in the poorest CPI coverage quartile reported consuming significantly greater gram weight (g), dietary fibre (g) and energy (kcal) as compared with those with the best coverage.

**Conclusions:**

Differential CPI price coverage exists among food components and commonly consumed food groups; additionally dietary intake differs significantly in the population by CPI coverage. Methodological refinements are needed to better account for error when using prices from food supply data to estimate diet costs.

The burden of diet-related diseases remains a substantial population health problem worldwide^(1)^. Within Canada, where we conducted the current study, poor diet is the third largest contributor to death and disability, accounting for an estimated 48 000 deaths and over 860 000 years of healthy life lost in 2016^(2)^.

The cost of food is a key influence on diet^(3–7)^. A growing literature has examined how diet cost predicts dietary intakes and diet quality^(3,5)^, including population-representative studies in the USA, Sweden and Spain, and cohort studies in the USA, UK and France^(8–12)^. To date, this literature suggests that higher diet costs tend to be associated with better dietary quality, as indicated by fruit and vegetable intake, energy density and holistic healthy eating measures^(5,8–13)^. Diet cost literature within Canada is highly limited, with no population-representative studies to date. Two Canadian studies have reported the prices of margarine and oils in relation to their nutritional value, and another examined the association between diet cost and quality in grade 5 children living in Alberta^(14–16)^. Some related research has examined expenditure patterns in a nationally representative sample of households, which can help to inform inferences about diet costs^(17)^. Together, these studies set a foundation for the modern advancement of diet costing and food pricing methodology in Canada, with a focus on price collection^(14–17)^.

The majority of diet cost research involves methodology matching intake data from population-based dietary surveillance surveys to food prices from pre-collected food supply data, such as national food price monitoring databases, rather than a proximal measure of individual/household spending collected contemporaneously or linked directly to respondent intake data records^(5,8–13,16)^. No studies have examined the measurement error introduced from using food supply prices in this way, and its potential influence on estimating diet costs. Therefore, the aim of the current study was to examine the coverage of dietary intakes reported in the 2015 Canadian Community Health Survey-Nutrition (CCHS-N) by the Canadian Consumer Price Index (CPI) to price dietary intakes and relatedly the nutritional significance of measurement error. In particular, we were interested in understanding the extent and quality of coverage of CPI food price data to provide prices for main food groups and food components that contribute to dietary intake. Our objectives were to (i) describe the coverage of the CPI food price data on the overall dietary intakes of Canadians, and by food components and food groups and (ii) describe mean nutrient intakes based on the degree of CPI coverage within individuals’ total diets.

## Methods

### Data sources

We matched 24-h dietary recall data from the 2015 CCHS-N to the 2015 Canadian CPI^(18,19)^. The 2015 CCHS-N is the most recent nationally representative dietary surveillance survey in Canada, administered by Statistics Canada^(18)^. In 2015, 20 487 respondents aged one and older, residing in private dwellings in one of the ten Canadian provinces completed the CCHS-N^(18)^. For respondents under 6 years old, a proxy (parent/guardian) completed the CCHS-N, for those between 6 and 11 years old, the survey was proxy-assisted, and those 12 years old and older completed the survey themselves^(18)^. All respondents completed a first 24 h-dietary recall along with a socio-demographic and health questionnaire^(18)^. A subset of respondents completed a second 24-h recall 3 to 10 d later^(18)^. Diet recalls were completed using the Automated Multiple-Pass Method^(18)^. In the 2015 CCHS-N, respondents reported intakes of over 2500 unique food items in the first 24-h dietary recall which was then combined with energy and nutrient information using the Canadian Nutrient File and collapsed into 156 Bureau of Nutritional Sciences food product categories^(18)^. The CCHS-N excludes individuals living in the territories, on reserves and other Aboriginal settlements, full-time members of the Canadian Forces and institutionalised populations^(18)^. The current analysis uses the reported intakes from the first recall only^(18)^.

The Canadian CPI, administered and maintained by Statistics Canada, collects and monitors monthly prices of consumer goods in eight aggregate categories, of which one is food^(19)^. The CPI publishes the percent change in monthly prices at various Census geographies and the average monthly retail price for select items provincially and/or nationally^(19)^. In 2015, national prices were collected for forty-nine food items through field collection or scanner data, and the average price of each product was weighted by the population area to derive an average Canadian retail price for that product^(19)^.

### Matching the Canadian Community Health Survey-Nutrition and Canadian Consumer Price Index

We matched each of the 156 Bureau of Nutritional Sciences food product categories, excluding the water category, to the 2015 CPI average retail items ($/g) as either a match or non-match. Where further product information was needed from the CCHS-N (e.g. ‘other fruit’ category) to match to the CPI, the most consumed (g) item within that food category reported from the CCHS-N was used. This process is consistent with CPI methodology where the representative product is selected based on consumer popularity^(20)^. We used mean gram weight for the food items consumed in the 2015 CCHS-N as a proxy for consumer popularity. Water was excluded from the current analysis as it could not be reliably estimated.

The process by which food items were matched was informed by the diet cost literature wherein foods are sometimes matched based on food group or a product type that might be reasonably substituted at retail purchase, as an exact match is not always possible^(5,8–13)^. One member of the team first attempted to find an exact match for items in the CCHS-N using the CPI. For example, ‘Beef: Ground’ (BNSD22C) in the CCHS-N was an exact match to ‘Ground Beef’ in the CPI. If an exact item match did not exist, then two members of the team worked together to find a near-exact match by selecting a comparable product considering the food group, price, and then finally the nutrient composition of the proposed match item, in that order. Some near-matches were straightforward, for example, ‘Sausage’ (BNSD30A) in the CCHS-N was matched to ‘Wieners’ in the CPI. Other matches involved more discussion by the team and reference to the literature, for example, for the three cheese products listed (BNSD 14B, 14C, 14D), we identified that the CPI item of processed cheese provided a comparable match despite the three different % BF ranges for each of the three products. A judgement was also made about the risk of bias introduced by having no match at all (e.g. using this criterion, margarine in the CCHS-N was matched to butter in the CPI). Exact and near-exact matches were coded as a match. If no comparable product was identified in that process, then it was coded as a non-match. For instance, all baked products were non-matches, as the closet match was bread in the CPI and that was not deemed a comparable product on the basis of food group, price or nutrient composition. Finally, all non-match items were re-evaluated once more by three members of the team, comparing to other diet cost literature. The final matches are described in Table 1. Altogether, despite the potential for overmatching (see limitations), this matching process is consistent with previous methods used in the diet cost literature^(12,21)^.

**Table 1:** Description of food groups and food items in the Canadian Community Health Survey-Nutrition (CCHS-N) 2015 by Bureau of Nutritional Sciences categories, matched to the 2015 Canadian Consumer Price Index (CPI) based on food group, price and nutrient composition

Food group	BNS food category	BNS Food code	2015 CPI food item
Additions	Sauces (White/Bearnaise/Soya/Tartar/Ketchup)	BNSD50D	Ketchup
	Others (Baking Soda/Baking Powder/Yeast)	BNSD53B	*
	Seasonings (Salt/Vinegar)	BNSD50F	*
	Gravies	BNSD50C	*
	Spices	BNSD53A	*
	Whipping cream	BNSD13A	*
	Table cream	BNSD13B	*
	Half and Half cream	BNSD13C	*
Fats and oils	Butter	BNSD17A	Butter
	Regular Margarine	BNSD18A	Butter
	Block Margarine	BNSD20A	Butter
	Calorie-Reduced Margarine	BNSD18B	Butter
	Vegetable oils	BNSD21A	Cooking or salad oil
	Animal Fats	BNSD21B	Butter
	Shortening	BNSD21C	Butter
	Salad Dressings (With or Without oil)	BNSD50E	Cooking or salad oil
Fruits	Citrus Fruit (Oranges/Lemons/Grapefruits)	BNSD40A	Oranges
	Apple	BNSD40B	Apples
	Banana	BNSD40C	Bananas
	Cherries	BNSD40D	*
	Grapes/Raisins	BNSD40E	*
	Melons (Cantaloupe/Honeydew/Watermelon)	BNSD40F	*
	Peaches/Nectarines	BNSD40G	*
	Pears	BNSD40H	*
	Pineapple	BNSD40I	*
	Plums/prunes	BNSD40J	*
	Strawberries	BNSD40K	*
	Other Fruits (Blueberry/Date/Kiwi/Fruit Salad)	BNSD40L	*
Vegetables	Carrots	BNSD36E	Carrots
	Celery	BNSD36F	Celery
	Mushrooms	BNSD36I	Mushrooms
	Onion/Green Onions/Leks/Garlic	BNSD36J	Onions
	Tomatoes	BNSD36N	Tomato, canned
	Fried/Roasted Potatoes	BNSD38B	French fried potatoes
	Potato	BNSD39A	Potatoes
	Beans	BNSD36A	*
	Broccoli	BNSD36B	*
	Cabbage/Kale	BNSD36C	*
	Cauliflower	BNSD36D	*
	Corn	BNSD36G	*
	Lettuce/Leafy greens (Spinach/Mustard Greens)	BNSD36H	*
	Peas/Snow Peas	BNSD36K	*
	Peppers: Red/Green	BNSD36L	*
	Squashes	BNSD36M	*
	Other Veg. (Cucumber/Beet/Turnip)	BNSD36P	*
Milk and dairy products	Milk: Whole	BNSD10A	Homogenised milk
	Milk: 2 %	BNSD10B	Partly skim
	Milk: 1 %	BNSD10C	Partly skim
	Milk: Skim	BNSD10D	Partly skim
	Milk: Evaporated Whole Milk	BNSD10E	Evaporated milk
	Milk: Evaporated 2 %	BNSD10F	Evaporated milk
	Milk: Evaporated Skim	BNSD10G	Evaporated milk
	Cheese: Less than 10 % B.F	BNSD14B	Processed cheese
	Cheese: 10 % BF to 25 % B.F	BNSD14C	Processed cheese
	Cheese: More than 25 % B.F	BNSD14D	Processed cheese
	Milk: Condensed	BNSD10H	*
	Milk: Other (Whey/Buttermilk)	BNSD10I	*
	Milk: Goat/Sheep	BNSD10K	*
	Sour cream	BNSD13D	*
	Cottage Cheese	BNSD14A	*
	Yoghurts: Less than 2 % B.F	BNSD15A	*
	Yoghurts: More than 2·1 % B.F	BNSD15B	*
Meat products	Chicken: Meat Only	BNSD27A	Chicken
	Chicken: Meat + Skin	BNSD27B	Chicken
	Pork: Fresh – Lean Only	BNSD25A	Pork chops
	Pork: Fresh – Lean + Fat/Ground	BNSD25B	Pork chops
	Bacon	BNSD25C	Bacon
	Ham: Cured – Lean Only	BNSD25D	Bacon
	Ham: Cured – Lean + Fat	BNSD25E	Bacon
	Beef: Ground	BNSD22C	Ground beef
	Beef: Lean only	BNSD22A	Sirloin steak
	Beef: Lean + Fat	BNSD22B	Sirloin steak
	Turkey: Meat Only	BNSD27C	*
	Turkey: Meat + Skin/Ground	BNSD27D	*
	Other birds: Duck/Pheasant/Pigeon	BNSD27E	*
	Birds: Skin only	BNSD27F	*
	Liver Pate	BNSD28B	*
	Offal	BNSD29A	*
	Liver	BNSD28A	*
	Game Meat	BNSD31A	*
	Lamb: Lean Only	BNSD24A	*
	Veal: Lean + Fat/Ground	BNSD23B	*
	Lamb: Lean + Fat/Ground	BNSD24B	*
	Veal: Lean Only	BNSD23A	*
Processed meats	Ham: Cured – Lean Only	BNSD25D	Bacon
	Ham: Cured – Lean + Fat	BNSD25E	Bacon
	Bacon	BNSD25C	Bacon
	Luncheon Meat	BNSD32A	Wieners
	Liver Pate	BNSD28B	*
Protein alternatives	Peanut Butter/Other Nut Spreads	BNSD33C	Peanut butter
	Egg	BNSD16A	Eggs
	Legume	BNSD37A	Baked beans, canned
	Foods made with Vegetable Proteins (Tofu)	BNSD37B	*
	Nuts	BNSD33A	*
	Seeds	BNSD33B	*
	Egg Substitutes	BNSD16B	*
Finfish and shellfish products	Fish: Less than 6 % Total Fat	BNSD34	Canned sockeye salmon
	Fish: Superior or Equal to 6 % Total Fat	BNSD34B	Canned sockeye salmon
	Shellfish	BNSD35A	*
Grains	Pasta	BNSD01A	Macaroni
	Cereal/Grains/Flours	BNSD01C	Flour
	White Breads	BNSD02A	Bread
	Whole Wheat Breads	BNSD03A	Bread
	Other Whole Grain Breads	BNSD03B	Bread
	Rolls/Bagels/Pita/Croutons/Dumplings/ Matzo/Tortilla	BNSD04A	Bread
	Crackers/Crispbreads	BNSD04B	Soda crackers
	Rice	BNSD01B	*
Baked products	Muffins/English muffins	BNSD04C	*
	Pancakes/Waffles	BNSD04D	*
	Croissants/Piecrust/Phyllo Dough	BNSD04E	*
	Dry Mixes (Cakes/Muffins/Pancakes)	BNSD04F	*
	Cookies: Commercial	BNSD07A	*
	Pies: Commercial	BNSD08A	*
	Cakes: Commercial (Frozen Cake)	BNSD08B	*
	Danishes/Doughnuts/Other Pastries: Commercial	BNSD08C	*
	Biscuits: Commercial	BNSD07B	*
Breakfast Cereals	Whole Grain/Oats/High Fibre Breakfast Cereals	BNSD05A	Corn flakes
	Breakfast Cereal (Other)	BNSD06A	Corn flakes
Sweets	Sugars: White/Brown	BNSD41A	White sugar
	Ice Cream	BNSD09A	*
	Ice Mlk	BNSD09B	*
	Frozen Yogurt	BNSD09C	*
	Jam/Jellies/Marmalade	BNSD41B	*
	Other sugars (Syrups/Molasses/Honey)	BNSD41C	*
	Sugar Substitutes	BNSD41D	*
	Ice pop/Sherbet	BNSD43B	*
	Gelatin/Dessert Toppings/Pudding Mixes: Commercial	BNSD43C	*
	Chocolate Bar	BNSD44A	*
	Candy/Gum	BNSD43A	*
Snacks	Potato Chips	BNSD38A	*
	Salty/High-Fat Snacks (incl Tortilla Chips)	BNSD42B	*
	Plain Popcorn/Pretzels	BNSD42A	*
	Granola Bar	BNSD07C	*
Entrees	Soups without Vegetables	BNSD50B	Soup
	Soups with Vegetables	BNSD50A	Soup
	Mexican Recipes	BNSD99A	*
	Meal Replacements	BNSD54C	*
	Energy Bar	BNSD54A	*
	Protein Bar/shake	BNSD54B	*
Non-alcoholic beverages	Fruit Juice	BNSD45A	Orange juice, tetra-brick
	Fruit Drinks	BNSD46C	Orange juice, tetra-brick; Apple juice, canned
	Juices: Tomato & Vegetables	BNSD36O	Tomato juice, canned
	Soft Drink: Regular	BNSD46A	Soft drinks, cola
	Soft Drink: Diet	BNSD46B	Soft drinks, cola
	Tea (incl Iced Tea)	BNSD51A	Tea
	Coffee	BNSD51B	Coffee, roasted
	Plant-based Beverage (Soy/Almond/Coconut)	BNSD10J	*
	Other Beverages (Malted Milk/Chocolate Beverage)	BNSD46D	*
	Energy Drink	BNSD46E	*
	Vitamin Water	BNSD46F	*
	Sports Drink	BNSD46G	*
Alcoholic beverages	Spirits	BNSD47A	*
	Liqueurs	BNSD47B	*
	Wine	BNSD48A	*
	Beer	BNSD49A	*
	Coolers	BNSD49B	*
Baby foods	Babyfood products	BNSD52A	Baby food
	Infant Formula	BNSD52B	Baby food

*No match available in the CPI.

The proportion of individuals’ total intakes covered by the CPI prices (matched items) was described as a percent of the gram weight of all foods and beverages consumed (excluding water), energy, macronutrients, total sugar, sodium and total dietary fibre, recognising the impact of these food components on non-communicable disease risk^(1)^. Additionally, to gain an understanding of CPI coverage in relation to food items, the proportion of foods consumed (g) within eighteen food groups with matches to CPI was calculated for each respondent^(1)^. Food groups were created based on Kirkpatrick and colleagues’ food groupings that were used to assess the top food sources of energy, sodium, sugars and saturated fats in Canadians’ diets^(22)^.

### Analyses

Proportions and means were generated to describe CPI coverage in relation to total intake (g), energy (kcal), macronutrients (g), total sugar (g), sodium (mg), total dietary fibre (g) and food groups (g). Quartiles of CPI coverage were then assigned based on the proportion of total individual intake (g) captured by the CPI. Group mean intakes of energy and food components were compared across quartiles, applying a Bonferroni correction to obtain a Bonferroni corrected *P*-value (*α* = 0·05).

The analysis was conducted on STATA/MP 16.1 statistical software. The CCHS-N Public Use Microdata File was accessed through the Data Liberation Initiative at Dalhousie University. Accompanying Statistics Canada sampling weights were used to create weighted point estimates which represented the 34·5 million Canadians based on the Canadian Dietary Reference Intake age-sex groups and the 2011 Canadian Census population size^(18)^. The Canadian Dietary Reference Intake age-sex groups refer to Health Canada groupings used to determine the recommended intakes of nutrients and energy for a healthy individual based on their age and sex^(23)^. Bootstrap replication weights (*B* 500) provided by Statistics Canada were used to generate 95 % CI that took into account the multi-stage survey design used by the CCHS-N^(18)^.

## Results

### Canadian Consumer Price Index coverage overall and by food groups and food components

For overall CPI coverage of dietary intakes, we found that sixty-seven of the 155 items (43·2 %) in the 2015 CCHS-N matched to food prices in the 2015 CPI, accounting for an average of 76·3 % (95 % CI: 75·9, 76·7) of total gram weight consumed by Canadians. A detailed description of the food groups, food items and matches to the CPI is presented in Table 1.

Table 2 shows the extent of CPI coverage by food groups and food components. We found that fats and oils, processed meats, breakfast cereals and baby food groups had the best CPI coverage among the eighteen food groups. Fats and oils were consumed by 92·9 % (95 % CI 92·2, 93·6) of Canadians on their recall day, processed meats were consumed by 37·3 % (95 % CI 35·9, 38·7), breakfast cereals were consumed by 32·2 % (95 % CI 31·0, 33·3) and baby foods were consumed by 0·6 % (95 % CI 0·4, 0·8). In contrast, baked products, alcoholic beverages and snack food groups had the poorest CPI coverage (Table 2). Baked products, alcoholic beverages and snack foods were consumed by 27·3 % (95 % CI: 26·1, 28·5), 21·9 % (95 % CI: 20·8, 23·0) and 26·4 % (95 % CI 25·3, 27·7) of the population on their recall day.

**Table 2:** Mean and median proportion of Canadians’ dietary intakes in Canadian Community Health Survey-Nutrition (CCHS-*N*) 2015 covered by the Canadian Consumer Price Index (CPI), by total grams of each food group and overall, adjusted for Canadian Dietary Reference Intake (DRI) age-sex groups^(23)^

Food group	Proportion of the population who consumed the food groupon their 1^st^ 24-h recall day		Mean proportion of CPI coverage		Median proportion of CPI coverage
	%	95 % CI	%	95 % CI	%
Overall	100·0		76·3	75·9, 76·7	79·2
Additions	93·1	92·5, 93·8	25·7	24·6, 26·8	0·0
Fats and oils	92·9	92·2, 93·6	100·0		100·0
Fruits	71·8	70·5, 73·0	56·0	54·7, 57·3	66·5
Vegetables	92·9	92·2, 93·6	60·1	59·3, 61·0	64·3
Milk and dairy products	91·6	90·8, 92·4	87·3	86·5, 88·1	100·0
Meat products	72·5	71·3, 73·6	93·7	93.1, 94.4	100·0
Processed meats	37·3	35·9, 38·7	99·7	99·6, 99·8	100·0
Protein alternatives	74·0	72·7, 75·3	81·5	80·6, 82·4	100·0
Finfish and shellfish products	17·3	16·3, 18·4	75·5	73·1, 78·0	100·0
Grains	96·0	95·5, 96·5	83·6	82·6, 84·6	100·0
Baked products	27·3	26·1, 28·5	0·0		0·0
Breakfast cereals	32·2	31·0, 33·3	100·0		100·0
Sweets	80·5	79·4, 81·6	44·8	43·6, 46·0	31·3
Snacks	26·4	25·3, 27·7	0·0		0·0
Entrees	28·6	27·5, 29·8	85·3	83·9, 86·9	100·0
Non-alcoholic beverages	90·2	89·5, 90·9	95·8	95·3, 96·4	100·0
Alcoholic beverages	21·9	20·8, 23·0	0·0		0·0
Baby food	0·6	0·4, 0·8	100·0		100·0

Examining the variation in coverage further, Table 2 shows the computed median proportion of CPI coverage among food groups, which indicates that the majority of the data is left skewed, suggesting that there are outliers with poor CPI match that are influencing the mean estimate.

In Canadian Dietary Reference Intake age-sex group adjusted analyses, the CPI coverage for gram weight, energy and food components ranged from an average of 65·8 % (95 % CI 65·2, 66·4) for sodium intake to 82·3 % (95 % CI 81·9, 82·7) for protein intake (Table 3). In Table 3, the estimates of gram weight, energy and nutrients of study are consistently left skewed, with median intakes ranging from 68·3 % for sodium intake to 87·4 % for protein intake.

**Table 3:** Mean and median proportion of Canadians’ dietary intakes covered by the Canadian Consumer Price Index (CPI), by gram weight, energy and food components adjusted for DRI age-sex groups, 2015

Gram weight, energy, and nutrients of study	Mean proportion of CPI coverage		Median (%)
	%	95 % CI	
Gram weight	76·3	75·9, 76·7	79·2
Energy	72·9	72·5, 73·3	75·3
Protein	82·3	81·9, 82·7	87·4
Fat	78·4	77·9, 78·8	83·7
Carbohydrates	68·9	68·4, 69·4	71·5
Total sugar	66·9	66·3, 67·5	69·3
Sodium	65·8	65·2, 66·4	68·3
Dietary fibre	67·2	66·6, 67·7	70·6

### Respondent energy and nutrient intakes by Canadian Consumer Price Index coverage

The mean intake of energy and food components for each quartile of the population, when stratified by total CPI coverage (gram weight captured by the CPI), is shown in Table 4. When comparing the proportion of each age group captured within quartile 1 and quartile 4, there were significant differences only for specific age groupings of children and adults, wherein there was a greater proportion of children, ages 6 to 12, and adults, ages 18 to 54, in quartile 1 as compared with quartile 4.

**Table 4:** Canadians’ dietary intakes of energy and food components by quartile of Canadian Consumer Price Index match, 2015

	Intakes captured by CPI, quartiles							
	1		2		3		4	
	Mean	95 %CI	Mean	95 %CI	Mean	95 %CI	Mean	95 %CI
Characteristics of each quartile created based on gram weight captured by the CPI (%)	54·9 Min: 0·0 Max: 68·3	54·3, 55·5	74·9 Min: 68·3 Max: 80·8	74·7, 75·1	85·4 Min: 80·8 Max: 89·8	85·3, 85·6	94·7 Min: 89·8 Max: 100·0	94·5, 94·8
Each age group† captured within each quartile (%)	Young children 23·2	20·2, 26·4	Young children 29·0	25·6, 32·6	Young children 28·8	25·0, 32·9	Young children 19·1	16·0, 22·5
	Children** 25·8	22·9, 29·0	Children 30·4	27·1, 33·8	Children 24·8	22·0, 27·9	Children 19·0	16·5, 21·7
	Adolescents 23·5	20·8, 26·4	Adolescents 23·9	20·6, 27·7	Adolescents 25·3	22·3, 28·6	Adolescents 27·3	23·9, 30·9
	Adults*** 30·3	28·6, 32·0	Adults 25·4	23·6, 27·2	Adults 22·7	21·0, 24·4	Adults 21·6	20·1, 23·2
	Older adults 25·4	23·5, 27·5	Older adults 25·4	23·6, 27·2	Older adults 23·8	22·0, 25·8	Older adults 25·4	23·7, 27·1
Total gram weight, g	1898·9*	1851·7, 1946·2	1836·2	1799·1, 1873·3	1812·4	1770·7, 1854·1	1800·2	1751·7, 1848·6
Total energy, kcal	1917·5***	1873·3, 1961·7	1927·0***	1879·2, 1974·9	1840·7**	1797·4, 1884·0	1746·4	1698·7, 1794·1
Energy intake captured by the CPI (%)	58·4***	57·6, 59·2	69·4***	68·8, 70·0	78·0***	77·5, 78·6	89·1	88·6, 89·6
Protein, g	77·6	75·7, 79·5	81·2***	78·6, 83·8	77·4	75·1, 79·8	74·1	71·8, 76·5
Protein intake captured by the CPI (%)	72·0***	71·0, 72·9	80·1***	79·3, 80·8	85·7***	85·1, 86·3	94·0	93·6, 94·3
Fat, g	67·6	65·4, 69·8	73·0***	70·7, 75·4	68·4	66·3, 70·4	65·8	63·5, 68·1
Fat intake captured by the CPI (%)	71·2***	70·1, 72·3	75·2***	74·2, 76·3	80·4***	79·6, 81·3	88·5	87·8, 89·2
Carbohydrates, g	224·3	219·0, 229·7	231·8**	225·9, 237·6	231·3**	225·7, 236·8	217·8	211·8, 223·8
Carbohydrate intake captured by the CPI (%)	53·2***	52·2, 54·1	64·7***	64·0, 65·3	74·4***	73·8, 75·1	87·2	86·7, 87·7
Total sugar, g	87·1	84·3, 89·9	94·0*	90·8, 97·2	95·1**	92·0, 98·2	87·6	84·3, 90·9
Total sugar intake captured by the CPI (%)	52·9***	51·6, 54·1	62·5***	61·6, 63·5	71·2***	70·3, 72·1	84·5	83·7, 85·3
Sodium, mg	2550·6**	2474·3, 2626·9	2655·3	2573·3, 2737·3	2725·4	2648·2, 2802·7	2756·7	2662·7, 2850·8
Sodium intake captured by the CPI (%)	59·0***	57·8, 60·2	63·9***	62·9, 64·9	68·4***	67·5, 69·3	73·4	72·4, 74·5
Dietary fibre, g	17·6***	17·1, 18·2	18·1***	17·6, 18·7	16·6***	16·0, 17·1	14·6	14·1, 15·0
Dietary fibre captured by the CPI (%)	53·6***	52·4, 54·8	62·6***	61·6, 63·5	71·3***	70·4, 72·2	84·6	83·9, 85·3

Comparison to quartile 4: *P < 0·05; **P < 0·01; ***P < 0·001.

† Age groups: Young children (1–5 years old), children (6–12 years old), adolescents (13–17 years old), adults (18–54 years old), older adults (55+ years old)^(23)^.

Respondents with the poorest quartile of CPI match (quartile 1) had a significantly greater mean intake of gram weight (g), energy (kcal) and dietary fibre (g) as compared with those with the best CPI match (quartile four). Conversely, those with the poorest CPI match had significantly lower mean sodium (mg) as compared with those with the best CPI match. Those with the poorest CPI match also had a significantly lower percentage CPI capture of energy, protein, fat, carbohydrates, total sugar, sodium and total dietary fibre as compared with those with the best CPI match (Table 4).

## Discussion

### Summary of results

We found that the 2015 Canadian CPI covered 76·3 % of Canadians’ total dietary intake by weight (g) without water as assessed in the 2015 CCHS-N. This overall extent of CPI coverage is comparable to that of a diet cost study using Statistics Sweden data which identified that food price data covered 71 % of food intakes^(9)^. CPI coverage among food groups was heavily left skewed with the mean and median CPI match ranging from 0·0 % to 100·0 %. Indeed, the majority of food groups in our analysis had a median of 100 % coverage, namely, many staple foods and food groups that are a priority to economic monitoring about the food supply. By gram weight, energy and food components, however, there was differential coverage, wherein protein intake was best covered by the CPI and sodium was the poorest covered.

Finally, in our analysis, we found that the poorest quartile of CPI capture occurred among individuals with high intakes of food by gram weight, energy and fibre. Those in the lowest quartile of CPI match also have a significantly lower percentage of their total diet priced for energy, protein, fat carbohydrates, total sugar, sodium and dietary fibre as compared with those with the best CPI match.

Together, these findings indicate that a majority of Canadians’ dietary intake, including many staple food groups and commonly consumed foods, is well covered by using CPI food supply data to price dietary intakes. Yet using CPI prices to estimate broader pricing patterns across diets and populations may result in under coverage bias and measurement error. These types of error have not yet been well quantified, nor their potential presence consistently described in the literature on diet cost methodology. Thus, our analysis suggests that the CPI is a fair starting point for pricing dietary intakes in Canada, particularly given the lack of more proximal measures and population-based data on what Canadians pay for what they consume. Yet, our coverage analysis also demonstrates that further research and refinement of price data sources for population-representative analyses of the effects of food price on diet is needed. The development of robust price data sources is especially important for specific food groups where CPI does not provide prices for all items within the 2015 CCHS-N. As we will discuss further below, a number of these food groups are of key concern to the burden of diet-related noncommunicable diseases risk, and differences in CPI coverage by food groups could result in differential misclassification bias when analysing the influence of diet costs on dietary intake. Moreover, our findings are illustrative that similar problems relating to differential coverage of food supply databases may affect the results and interpretation of existing diet cost analyses in the literature. These sources of error have neither been comprehensively described nor consistently evaluated in diet cost research to date.

### Canadian Consumer Price Index coverage for staple foods, *v*. undercoverage for sweets and snacks

On average, staple food groups that were more commonly consumed within the population had better CPI price coverage than less commonly consumed food groups. Products including grains (96·0 %), fats and oils (92·9 %), milk and dairy products (91·6 %) and meat products (72·5 %) were food groups consumed by the population, and the mean proportion of CPI coverage in these food groups ranged from 83·6 % for grains to 100·0 % for fats and oils. The median proportion of CPI coverage for all of these items was 100·0 %. In these cases, the CPI proves to be a useful measure to cost commonly consumed food group items.

Our analysis showed that several other commonly consumed food groups, however, were not at all well covered by CPI prices. We found that vegetables (92·9 %), additions (93·1 %) and sweet (80·5 %) food groups that were similarly commonly consumed had CPI coverage by food group of just 60·1 %, 25·7 % and 44·8 %, respectively. Furthermore, among all food groups, those with < 50 % CPI coverage were snack foods, baked products, sweets, additions and alcoholic beverages.

A recent analysis of 2015 CCHS-N by Kirkpatrick and colleagues identified that foods such as muffins, quick breads, biscuits, dairy dessert, chocolate and cookies were among the top twenty contributors to energy, sodium, saturated fat and sugar intake^(22)^. These food items fall precisely within the food groups poorly covered in price data by the CPI^(24–27)^. Hence, the poor coverage of food groups of nutritional concern could result in error when pricing diets and studying the association between diet costs and dietary intake, with implications for our understanding of how food price affects diet quality and consequently diet-related diseases risk.

### Could we simply use other available price data to fill the gaps?

Our analysis highlights the need to fill the gaps in dietary coverage by CPI. In the absence of purpose-built data sets to price dietary intakes in our context in Canada, a potential solution for addressing these gaps in price coverage is to use other supermarket audit or scanner data for food groups not well covered. However, these approaches may also introduce error in analysis and interpretation resulting from heterogeneity in price data collection^(12,28,29)^. Another potential source of regional food price data in Canada is the National Nutritious Food Basket, collected annually by provinces and territories^(30)^. The National Nutritious Food Basket is a surveillance tool used to assess the cost and affordability of healthy eating in a given year, based on a market basket composed of 60–70 indicator food items selected to be consistent with Canada’s Food Guide and commonly consumed foods as measured in CCHS-N intake data^(30–33)^. Briefly, the regional National Nutritious Food Basket presents substantial limitations for population-based diet costing work^(31–33)^. Sub-national jurisdictions have discretion to modify the design and implementation of the food costing method including store selection, community inclusion, food price calculation and food item inclusion for regional purposes. Furthermore, neither comprehensive regional food price lists nor item lists are typically publicly shared, although many governments do make them available upon request. Like the CPI, the National Nutritious Food Basket does not collect food prices for snacks and sweet foods and thus could not be used to fill in price gaps for missing CPI products.

Additionally, in the USA in 2008, the United States Department of Agriculture (USDA) introduced a cross-sectional food price list intended for use with analyses of dietary data in 2008^(34)^. The national food price database was designed to correspond to the dietary intakes of the 2001–2002 National Health and Nutrition Examination Survey and was updated once for the 2003–2004 National Health and Nutrition Examination Survey^(4,34)^. One well-designed diet cost study among USA women identified that of the 467 food products in reported intakes, just twenty-seven items were not easily matched to the USDA food price database^(12)^. In this example, Bernstein and colleagues identified the process of obtaining the prices of their remaining twenty-seven unmatched items which included pricing these items from retail food outlets^(12)^.

In our analysis, eighty-eight of the 155 items were not initially easily matched, although they accounted for a minority of dietary intake by gram weight. To estimate diet costs, the prices of the remaining eighty-eight items could be derived using Bernstein’s method for missing items. However, the analysis in the present paper highlights that CPI coverage varies by food group, with processed meats and fats and oil having better CPI coverage than snacks and baked products for example. Therefore, it is clear that attention should be given to identifying the quantity, type and nutritional quality of unmatched food items to assess potential biases that may be introduced when estimating diet costs and the association to dietary intake.

Another issue in using heterogeneous sources for price data in diet cost estimation is that different methodological standards have been developed for measurement of prices in different nutrition sub-fields. Collecting price information from multiple sources can increase the specificity of price data for diet cost analyses. For instance, the missing food supply data in diet cost studies have often been addressed using supermarket audit data^(12,28)^. Other studies purposefully combine price data from multiple sources including through market research and CPI measures^(12,29)^.

For example, Rehm and colleagues (2011) assessed diet costs among American adults from 2001 to 2002 and used the aforementioned USDA food price database as the main source^(21)^. Where prices for alcoholic beverages, namely beer, wine, whisky and vodka were not available through the USDA database, they were available through the American CPI^(21)^. In this case, the researchers took into account the systematic error due to the difference purposes of each database when supplementing the USDA database with alcohol prices from the CPI^(21)^. In order to correct for this potential error, a subset of foods – fresh celery, ground coffee and white bread prices – were compared between the USDA database and the 2001–2002 CPI^(21)^. The researchers found that the prices were 10·4 % lower per edible portion in the database as compared with the CPI data^(21)^. Hence, the prices of alcoholic beverages in the CPI were deflated accordingly^(21)^.

As a final example, in retail food audits, the least expensive, smallest package size of the audited food item is often selected^(35,36)^. In contrast, food supply databases such as the CPI typically select an average price, average package size^(19)^. As such, retail audit prices at the population level used to price intakes may skew high in comparison with food supply prices, since many consumers will have actually paid a great deal less for the same volume/weight of food than the measured retail smallest package price. Certain subpopulations of consumers may be more sensitive to volume discounts, as well as volume discounts for certain food groups^(37, 38)^. Volume discounts are also more prevalent in certain types of food environments such as hypermarket stores^(38–41)^.

### Recommendations for food price data in diet cost analyses moving forward

The current study thus highlights four considerations to aid in future diet cost methodology and its interpretation. First, diet cost research should consistently quantify and report the extent and quality of match between food supply prices and intake data used. In the literature to date, the range of numbers of prices typically used for diet cost analysis are wide and the proportion of match is unclear. Identifying and reporting the details about how dietary intakes are priced allows for the assessment of potential biases that may be introduced.

Second, researchers should examine alternatives to using a single source of food supply data, beyond addressing missing values. Comparing different methods to derive diet costs can improve the estimation of diet costs and the examination of the association with dietary intakes.

Third, food supply data sets can present a risk of misclassification bias in matching intake data to estimate diet costs. For example, in the CPI, we found greater differential product coverage in milk and egg and beef products, in comparison with non-alcoholic beverages, fruits and vegetable products (Table 1). This differential coverage could influence the costing of specific food items/food groups and contribute to differential misclassification bias in attributing dietary costs to subpopulations. Publishing consistent methods to assign product matches from food price databases to dietary intakes could help to identify such potential biases in diet costing.

Fourth, researchers can consider the potential for systematic error, where prices for certain food categories are omitted altogether. For instance, the CPI did not provide any price information for alcoholic beverages despite alcoholic beverages being among the top contributors to energy intake within beverages in the 2015 CCHS-N^(26)^. In the 2015 CCHS-N, alcoholic beverages were consumed in the past 24 h by 21·8 % of respondents which accounted for an average of 3·2 % of total 24-h energy intake and 4·5 % of total intake based on total grams consumed. Diet costs analyses in the UK, USA and Japan identified that diet costs were positively associated with alcohol consumption among nationally representative samples of adults^(8,28,42)^. Bernstein and colleagues (2010) in the USA have been one of the few diet cost studies to describe the inclusion of alcoholic beverages^(12)^; in their case, they supplemented their main food supply price data set with retail audit data for remaining food items, including alcoholic beverages^(12)^. Hence, some consideration should be given to the inclusion/exclusion of certain categories, such as alcoholic beverages, given the potential contribution to dietary intake and diet costs.

### Strengths and limitations

This paper presents an assessment of measurement error to inform diet cost analysis based on Canada’s only nationally representative dietary intake surveillance data source. There are three main limitations. First, food items in the CPI are most consistent with the level of detail presented in the CCHS-N BNS groups which are already collapsed into 156 food categories, from over 2500 unique food items reported as consumed. The use of the CPI limited our ability to price all 2500 food items. Additionally, some items in the CCHS-N that were matched with CPI items were not an exact match, but instead a comparable match despite differences in food group, price or nutrient profiles (e.g. matching margarine from the CCHS-N to butter in the CPI). Hence, the CPI coverage estimates may be an overestimation of the food price information that is currently available in Canada to match to the CCHS-N^(43)^. This may also influence the association between diet costs and dietary intake or inferences about diet costs and health outcomes because some food items that were nutritionally different in the CCHS-N were matched to the same CPI item. Similarly, we further aggregated the 156 food categories into eighteen food groups which may hinder our understanding of CPI coverage of specific food items within the CCHS-N. Second, most diet cost methodology usually makes the assumption that intakes reported in dietary surveillance are purchased through retail food outlets and that sources of food supply price data are representative of what is available and accessible to consumers at retail. For example, within Canada, approximately 70 cents of every household food dollar is spent in a retail food store^(44)^. Food prices within the CPI are collected from retail food outlets, while dietary intake data in the CCHS-N recorded food items that were both purchased from retail food outlets and restaurant/quick service settings (a variable for location of purchase does not exist in this data set). We may have artificially overestimated the CPI coverage and underestimated food prices in instances where a substantial proportion of individuals’ intake comes from non-retailed foods. Third, the dietary intake data that were matched to the CPI were taken from a single 24-h dietary recall per respondent. A single 24-h recall provides rich detail about dietary intake on the reported day and is sufficient to estimate group means^(45)^. However, it should be noted that the current analysis does not account for episodically consumed foods. Addressing this was not necessary in the current analysis, but statistical methods have been developed to estimate usual intakes including episodically consumed items where it is needed to assess compliance with guidelines or correlate intakes with health outcomes^(46–48)^.

## Conclusion

Given the proliferation of diet costs studies using food supply prices over the last 20 years, the current study highlights potential sources of error and bias in using food supply data to price dietary intakes^(5)^. Researchers and decision makers should pursue opportunities for tailored data sets to price dietary intakes when using food supply data prices alone or in combination with other sources (e.g. retail audit data).
