# Feasibility and measurement error in using food supply data to estimate diet costs in Canada – ERRATUM

**DOI:** 10.1017/S1368980024001113

**Published:** 2024-05-30

**Authors:** Gabriella Luongo, Valerie Tarasuk, Yanqing Yi, Catherine L Mah

In the above article there are errors in Table 1. Within the Meat products food group, the BNS food category lines for Bacon, Ham: Cured – Lean Only, Ham: Cured – Lean + Fat, and Liver Pate should be removed. In the Processed meats food group, Sausage (BNSD30A) matched to Wieners in the CPI should be added.

**Table 1:** Description of food groups and food items in the Canadian Community Health Survey-Nutrition (CCHS-N) 2015 by Bureau of Nutritional Sciences categories, matched to the 2015 CPI based on food group, price, and nutrient composition

Food group	BNS food category	BNS Food code	2015 CPI food item
Additions	Sauces (white/Bearnaise/soya/tartar/ketchup)	BNSD50D	Ketchup
	Whipping cream	BNSD13A	*
	Table cream	BNSD13B	*
	Half and Half cream	BNSD13C	*
	Gravies	BNSD50C	*
	Seasonings (salt/vinegar)	BNSD50F	*
	Spices	BNSD53A	*
	Others (baking soda/baking/powder/yeast)	BNSD53B	*
Fats and oils	Butter	BNSD17A	Butter
	Regular Margarine	BNSD18A	Butter
	Calorie- Reduced Margarine	BNSD18B	Butter
	Block Margarine	BNSD20A	Butter
	Animal Fats	BNSD21B	Butter
	Shortening	BNSD21C	Butter
	Vegetable oils	BNSD21A	Cooking or salad oil
	Salad dressings (with or without oil)	BNSD50E	Cooking or salad oil
Fruits	Citrus Fruit (oranges/lemons/grapefruits)	BNSD40A	Oranges
	Apple	BNSD40B	Apples
	Banana	BNSD40C	Bananas
	Cherries	BNSD40D	*
	Grapes/raisins	BNSD40E	*
	Melons (cantaloupe/Honeydew/watermelon)	BNSD40F	*
	Peaches/nectarines	BNSD40G	*
	Pears	BNSD40H	*
	Pineapple	BNSD40I	*
	Plums/prunes	BNSD40J	*
	Strawberries	BNSD40K	*
	Other fruits (blueberry/Date/Kiwi/fruit salad)	BNSD40L	*
Vegetables	Carrots	BNSD36E	Carrots
	Celery	BNSD36F	Celery
	Mushrooms	BNSD36I	Mushrooms
	Onion/green onions/leeks/garlic	BNSD36J	Onions
	Tomatoes	BNSD36N	Tomato, canned
	Fried/Roasted Potatoes	BNSD38B	French fried potatoes
	Potato	BNSD39A	Potatoes
	Beans	BNSD36A	*
	Broccoli	BNSD36B	*
	Cabbage/Kale	BNSD36C	*
	Cauliflower	BNSD36D	*
	Corn	BNSD36G	*
	Lettuce/Leafy greens (spinach/mustard greens)	BNSD36H	*
	Peas/snow peas	BNSD36K	*
	Peppers: red/green	BNSD36L	*
	Squashes	BNSD36M	*
	Other veg. (cucumber/beet/turnip)	BNSD36P	*
Milk and dairy products	Milk: Whole	BNSD10A	Homogenized milk
	Milk: 2%	BNSD10B	Partly skim
	Milk: 1%	BNSD10C	Partly skim
	Milk: Skim	BNSD10D	Partly skim
	Milk: Evaporated Whole Milk	BNSD10E	Evaporated milk
	Milk: Evaporated 2%	BNSD10F	Evaporated milk
	Milk: Evaporated Skim	BNSD10G	Evaporated milk
	Cheese: Less than 10% BF	BNSD14B	Processed cheese
	Cheese: 10% BF to 25% BF	BNSD14C	Processed cheese
	Cheese: More than 25% BF	BNSD14D	Processed cheese
	Milk: Condensed	BNSD10H	*
	Milk: Other (Whey/Buttermilk)	BNSD10I	*
	Milk: Goat/Sheep	BNSD10K	*
	Sour Cream	BNSD13D	*
	Cottage Cheese	BNSD14A	*
	Yoghurts: Less than 2% BF	BNSD15A	*
	Yoghurts: More than 2.1% BF	BNSD15B	*
Meat products	Beef: Lean only	BNSD22A	Sirloin steak
	Beef: Lean + Fat	BNSD22B	Sirloin steak
	Beef: Ground	BNSD22C	Ground beef
	Pork: Fresh - Lean only	BNSD25A	Pork chops
	Pork: Fresh - Lean + Fat/ground	BNSD25B	Pork chops
	Chicken - meat only	BNSD27A	Chicken
	Chicken: Meat + skin	BNSD27B	Chicken
	Veal: Lean only	BNSD23A	*
	Veal: Lean + Fat/Ground	BNSD23B	*
	Lamb: lean only	BNSD24A	*
	Lamb: Lean + Fat/ground	BNSD24B	*
	Turkey: meat only	BNSD27C	*
	Turkey: Meat + skin/ground	BNSD27D	*
	Other birds: Duck/Pheasant/Pigeon	BNSD27E	*
	Birds: Skin only	BNSD27F	*
	Liver	BNSD28A	*
	Offal	BNSD29A	*
	Game Meat	BNSD31A	*
Processed meats	Bacon	BNSD25C	Bacon
	Ham: Cured - lean only	BNSD25D	Bacon
	Ham: Cured - lean + fat	BNSD25E	Bacon
	Sausage	BNSD30A	Wieners
	Luncheon Meat	BNSD32A	Wieners
	Liver Pate	BNSD28B	*
Protein alternatives	Egg	BNSD16A	Eggs
	Peanut butter/other nut spreads	BNSD33C	Peanut butter
	Legume	BNSD37A	Baked beans, canned
	Egg Substitutes	BNSD16B	*
	Nuts	BNSD33A	*
	Seeds	BNSD33B	*
	Foods made with vegetable proteins (tofu)	BNSD37B	*
Finfish and shellfish products	Fish: Less than 6% total fat	BNSD34	Canned sockeye salmon
	Fish: superior or equal to 6% total fat	BNSD34B	Canned sockeye salmon
	Shellfish	BNSD35A	*
Grains	Pasta	BNSD01A	Macaroni
	Cereal/Grains/Flours	BNSD01C	Flour
	White breads	BNSD02A	Bread
	Whole wheat breads	BNSD03A	Bread
	Other Whole Grain Breads	BNSD03B	Bread
	Rolls/bagels/pita/croutons/dumplings/ matzo/tortilla	BNSD04A	Bread
	Crackers/Crispbreads	BNSD04B	Soda crackers
	Rice	BNSD01B	*
Baked products	Muffins/English muffins	BNSD04C	*
	Pancakes/Waffles	BNSD04D	*
	Croissants/Piecrust/Phyllo Dough	BNSD04E	*
	Dry Mixes (Cakes/Muffins/Pancakes)	BNSD04F	*
	Cookies: Commercial	BNSD07A	*
	Biscuits: Commercial	BNSD07B	*
	Pies: Commercial	BNSD08A	*
	Cakes: Commercial (Frozen Cake)	BNSD08B	*
	Danishes/Doughnuts/Other Pastries: Commercial	BNSD08C	*
Breakfast Cereals	Whole Grain/Oats/High Fibre Breakfast Cereals	BNSD05A	Corn flakes
	Breakfast Cereal (other)	BNSD06A	Corn flakes
Sweets	Sugars: white/brown	BNSD41A	White sugar
	Ice Cream	BNSD09A	*
	Ice Milk	BNSD09B	*
	Frozen Yogurt	BNSD09C	*
	Jam/Jellies/Marmalade	BNSD41B	*
	Other sugars (syrups/molasses/honey)	BNSD41C	*
	Sugar substitutes	BNSD41D	*
	Candy/gum	BNSD43A	*
	Ice pop/Sherbet	BNSD43B	*
	Gelatin/Dessert Toppings/Pudding Mixes: Commercial	BNSD43C	*
	Chocolate Bar	BNSD44A	*
Snacks	Granola Bar	BNSD07C	*
	Potato Chips	BNSD38A	*
	Plain popcorn/pretzels	BNSD42A	*
	Salty/high-fat snacks (incl Tortilla chips)	BNSD42B	*
Entrees	Soups without vegetables	BNSD50B	Soup
	Soups with Vegetables	BNSD50A	Soup
	Energy Bar	BNSD54A	*
	Protein Bar/shake	BNSD54B	*
	Meal replacements	BNSD54C	*
	Mexican Recipes	BNSD99A	*
Non-alcoholic beverages	Juices: tomato & vegetables	BNSD36O	Tomato juice, canned
	Fruit Juice	BNSD45A	Orange juice, tetra-brick
	Soft Drink: Regular	BNSD46A	Soft drinks, cola
	Soft Drink: Diet	BNSD46B	Soft drinks, cola
	Fruit Drinks	BNSD46C	Orange juice, tetra-brick; Apple juice, canned
	Tea (incl iced tea)	BNSD51A	Tea
	Coffee	BNSD51B	Coffee, roasted
	Plant-based beverage (Soy/Almond/Coconut)	BNSD10J	*
	Other beverages (malted milk/chocolate beverage)	BNSD46D	*
	Energy Drink	BNSD46E	*
	Vitamin Water	BNSD46F	*
	Sports Drink	BNSD46G	*
Alcoholic beverages	Spirits	BNSD47A	*
	Liqueurs	BNSD47B	*
	Wine	BNSD48A	*
	Beer	BNSD49A	*
	Coolers	BNSD49B	*
Baby foods	Baby food products	BNSD52A	Baby food
	Infant formula	BNSD52B	Baby food

* No match available in the CPI

The correct table is below:
